# Short-term exposure to predation affects body elemental composition, climbing speed and survival ability in *Drosophila melanogaster*

**DOI:** 10.7717/peerj.2314

**Published:** 2016-08-04

**Authors:** Indrikis Krams, Sarah Eichler Inwood, Giedrius Trakimas, Ronalds Krams, Gordon M. Burghardt, David M. Butler, Severi Luoto, Tatjana Krama

**Affiliations:** 1 Department of Psychology, University of Tennessee, Knoxville, United States; 2 Institute of Ecology and Earth Sciences, University of Tartu, Tartu, Estonia; 3 Department of Risk Assessment and Epidemiology, Institute of Food Safety, Animal Health and Environment BIOR, Riga, Latvia; 4 Bredesen Center, Energy Science and Engineering, University of Tennessee, Knoxville, United States; 5 Centre for Ecology and Environmental Research, Vilnius University, Vilnius, Lithuania; 6 Department of Biotechnology, Daugavpils University, Daugavpils, Latvia; 7 Departments of Psychology and Ecology & Evolutionary Biology, University of Tennessee, Knoxville, TN, United States; 8 Department of Plant Sciences, University of Tennessee, Knoxville, United States; 9 School of Psychology, University of Auckland, Auckland, New Zealand; 10 English, Drama and Writing Studies, University of Auckland, Auckland, New Zealand; 11 Department of Plant Protection, Estonian University of Life Science, Tartu, Estonia

**Keywords:** *Drosophila melanogaster*, Fear ecology, Negative geotaxis, Elemental composition, Survival, Spider predation, Stress, Body reserves

## Abstract

Factors such as temperature, habitat, larval density, food availability and food quality substantially affect organismal development. In addition, risk of predation has a complex impact on the behavioural and morphological life history responses of prey. Responses to predation risk seem to be mediated by physiological stress, which is an adaptation for maintaining homeostasis and improving survivorship during life-threatening situations. We tested whether predator exposure during the larval phase of development has any influence on body elemental composition, energy reserves, body size, climbing speed and survival ability of adult *Drosophila melanogaster*. Fruit fly larvae were exposed to predation by jumping spiders (*Phidippus apacheanus*), and the percentage of carbon (C) and nitrogen (N) content, extracted lipids, escape response and survival were measured from predator-exposed and control adult flies. The results revealed predation as an important determinant of adult phenotype formation and survival ability. *D. melanogaster* reared together with spiders had a higher concentration of body N (but equal body C), a lower body mass and lipid reserves, a higher climbing speed and improved adult survival ability. The results suggest that the potential of predators to affect the development and the adult phenotype of *D. melanogaster* is high enough to use predators as a more natural stimulus in laboratory experiments when testing, for example, fruit fly memory and learning ability, or when comparing natural populations living under different predation pressures.

## Introduction

Developmental plasticity refers not only to the ability of an individual to modify its development in response to changing environmental conditions but, as evolutionary theory suggests, it might also facilitate the evolution of novel traits (Müller & Wagner, 1991; Moczek et al., 2011; Dias & Ressler, 2014). Predator risk can affect the behaviour and the physiology of prey by inducing stress (Wingfield et al., 1998), by increasing metabolic requirements (Beckerman, Wieski & Baird, 2007; Slos & Stoks, 2008) and by forcing energy allocation away from the processes of growth and development to survival (McPeek, 2004; Trussell, Ewanchuk & Matassa, 2006). These environmental effects can be explained by developmental plasticity in response to fluctuating environmental conditions. Predation is therefore an important factor in revealing the mechanisms of developmental plasticity in mediating the initiation and subsequent elaboration of incipient novel traits (Hõrak, Tummeleht & Talvik, 2006; Siepielski, Wang & Prince, 2014). Many studies differentiate between the lethal and nonlethal effects of the risk of predation on prey (see Lima & Dill, 1990 for a review). These nonlethal or nonconsumptive effects result from the perceived threat of predation and are often referred to as ‘the ecology of fear’ (Preisser & Bolnick, 2008; Sitvarin & Rypstra, 2014).

Recent research has revealed long-term effects caused by predators on the evolution of phenotypic traits in *D. melanogaster* (Lehmann, Goldman & Dworkin, 2013; O’Donnell et al., 2014). The presence of predators is known to create changes in a prey’s morphology (McCollum & Leimberger, 1997; Hossie et al., 2010) and selection in a prey’s phenotype on factors affecting escape ability (O’Steen, Cullum & Bennet, 2002; Janssens & Stoks, 2014). For example, a number of studies show a positive relationship between the risk of predation and the amount of fat reserves, an important determinant of reproductive success and survival (Muehlenbein & Bribiescas, 2005; Koskimäki et al., 2004; Krams et al., 2010; Rogers, 2015). Because predator–prey interactions are size-dependent, predation also has a strong influence on prey body sizes (Gooding & Harley, 2015; Peckarsky et al., 2008). Thus, the presence of predators in communities and the resulting non-consumptive ‘fear’ effects (Peckarsky et al., 2008; Siepielski, Wang & Prince, 2014) change the composition of such body elements as carbon (C) and nitrogen (N), energy reserves and the body size of prey, which should inevitably influence their survival strategies and anti-predator responses. Importantly, breaking down proteins may affect muscle mass and the ability to escape the predator (Bradley, Kelleher & Kimball, 2013). In grasshoppers, for example, predator presence impairs development by breaking down body proteins. This decreases body nitrogen and increases body carbon, thus ensuring the production of glucose and efficient anti-predator responses such as hiding behaviour (Hawlena & Schmitz, 2010a; Hawlena & Schmitz, 2010b; Hawlena et al., 2012). While these findings are induced by long-term predator presence, studies on the effects of short-term exposure are generally missing, which is one of the reasons that the present study employed an exposure time spanning only the larval stage.

In many arthropods, negative geotaxis is a frequently used index of locomotor behaviour. This startle-induced vertical movement constitutes an efficient response to avoid predators (Ladau, 2003; Médoc & Beisel, 2011), and is commonly assessed in *Drosophila* research (Ali et al., 2011; Morrow et al., 2004). Negative geotaxis is an innate escape reaction during which a fly climbs vertically after being tapped to the bottom of a vial. This response is estimated by either the distance an insect is able to ascend in a set time or the length of time it takes a fly to walk a set distance (Linderman et al., 2012). This behavioural assay serves as a reliable indicator of senescence and infection status in escape-related responses (Rhodenizer et al., 2008; Linderman et al., 2012). However, since negative geotaxis has never been studied as the escape response of fruit flies under the risk of predation, we tested whether the presence of spiders affects the phenotypic development of *Drosophila melanogaster*, and what role the altered body composition has in the performance of escape response. More specifically, we studied whether predator exposure during the larval stage affects the concentration of body N and C, body mass, fat reserves and climbing speed in a negative geotaxis test of adult *D. melanogaster*. ‘Fear’ ecology predicts lower body N and higher body C content, smaller adult sizes and fat reserves due to stressful conditions, and slower climbing speed in negative geotaxis tests, because decline in N should be associated with muscle mass (Hawlena & Schmitz, 2010a; Hawlena & Schmitz, 2010b; Hawlena et al., 2012). We also tested whether fruit flies exposed to predation at the larval stage have better chances to survive as adults. According to the general predictions of ‘fear’ ecology, the survival of adults should be lower in the flies exposed to predators because of their lower body N and slower climbing ability.

## Methods

### Animals and treatment groups

The stock animals were maintained in the lab of the University of Tennessee-Knoxville at 25 ± 1 °C under a constant 12:12 h light–dark cycle. The wild strain Oregon-R-modENCODE (#25211) of *D. melanogaster* were used as prey, while wild adult jumping spiders (*Phidippus apacheanus*) were used as predators. This spider species is distributed across the US (except for a few northern states) and prefers both larvae and adult moths and flies (Edwards, 1980). The fruit flies were obtained from Bloomington Drosophila Stock Center (IN, US). The spiders were collected in Florida, US, and were received from the supplier phids.net. This spider is easy to house and breed in captivity (see phids.net for more information).

The flies were isolated under carbon dioxide anesthesia. To ensure virginity, we isolated females within 5–7 h after imaginal eclosion, and placed them in groups of ten females and ten males per vial (9-cm height × 2.4-cm diameter) with 6 ml of food (cornmeal, dextrose and yeast medium) for 24 h. The adults were subsequently removed. When the eggs began to hatch, we placed fruit flies in Plexiglas jars (10 cm height × 12 cm diameter). In the experimental group, each of the jars contained one *P. apacheanus* spider. The vials were placed horizontally on the floor of the jar. The predators often walked into the vials, and we observed a number of attacks by *P. apacheanus* on the *D. melanogaster* larvae. As soon as the larvae started to pupate, the predators were removed from the jars. Thus, *D. melanogaster* individuals were exposed to the presence and direct consumption by *P. apacheanus* spiders only during the larval phase. In total, we had 20 experimental jars where *D. melanogaster* larvae were reared with spiders, and 20 control jars containing no spiders. The initial density of larvae was equal across the jars. We picked the first-instar larvae from the surface of the culture medium with a squirrel-brush and removed them so that each vial contained fixed densities of 150 larvae/vial which is considered to be average density for this vial size (Bierbaum, 1989).

### Fruit fly body C and N content

Within 9–10 h after imaginal eclosion, thirty randomly chosen males and thirty females from each jar (in total 1,200 flies of the experimental predator exposure group, and 1,200 individual flies of the control group) were placed separately in vials without food for 4 h with only water provided. This ensured all consumed food and faeces were released during the fasting period. Then the flies were dried at 75 °C for 72 h, and weighed as groups of 10 males and 10 females (Vijiendravarma, Narasimha & Kawecki, 2011) using a Sartorius MC5 microanalytical balance with an accuracy of ±1 µg. Dry body weight was calculated for individual flies as the dry weight of each replicate divided by the number of flies assigned in each replicate.

The percentage of C and N content was measured from the mass of whole flies using a C/N auto-analyser (Hawlena & Schmitz, 2010a; Hawlena & Schmitz, 2010b). Samples of C and N concentrations were measured as groups of 10 fruit flies equally representing each jar. In total, we measured 19 groups of males and 21 groups of females in the experimental group, as well as 20 groups of males and 19 groups of females as controls.

### Lipid extraction

Lipids were extracted with a combination of chloroform, methanol and water (Folch, Lees & Sloane-Stanley, 1957; Reis et al., 2013; Ren et al., 2014). Briefly, dried flies were ground into powder and eluted by chloroform and methanol (1:2, v/v). The samples were added with chloroform and water, vigorously vortexed and centrifuged. The organic phase was pipetted into a new glass tube into which chloroform was added. Four hours later, the mixture evaporated using a rotary evaporator. We extracted lipids in groups of 20 individuals for each sex (20 male and 20 female replicates in the experimental group, and 20 male and 20 female replicates in the control group equally representing each jar). The absolute quantity of body lipid for individual flies was calculated by dividing the lipid content (dry weight minus lipid-free dry weight) by the number of individuals assigned to each replicate. Prior to the experiments, we found that the repeatability measure of our analyses was high (*r* = 0.91, *P* < 0.001).

### Negative geotaxis assay

Climbing speed was measured for groups of ten individuals of the same sex (25 male and 25 female replicates in the experimental group, and 25 male and 25 female replicates in the control group). To avoid senescence-related effects in climbing speed of startled flies (Rhodenizer et al., 2008), negative geotaxis was measured within 2–3 days of eclosion. Sexes were sorted under carbon dioxide anesthesia and placed in a separate vial (9 cm height × 2.4 cm diameter). The flies were allowed 45 min to recover from anesthesia. We prepared the climbing apparatus so that two polystyrene vials were vertically joined by tape facing each other (Ali et al., 2011). We ensured that the openings of the vials were perfectly aligned with each other to provide an even climbing surface for the flies. For the lower vial, a vertical distance of 7 cm above the bottom surface was measured and each vial was marked by drawing a circle around the entire circumference of the vial. During each trial, we gently tapped the flies down to the bottom of the vial and measured the number of flies that can climb above the 7-cm mark by 10 s after the tap. The assay was repeated for the same group ten times, allowing for a 3-min rest period between each trial. We recorded the proportion of flies per group that passed the 7-cm mark.

### Survival

To test whether larval exposure to predators enhances survival during adulthood, we placed groups of 10 experimental and 10 control individuals in Plexiglas jars (10 cm height × 12 cm diameter) for 12 h during daylight time. Each jar contained one young adult *P. apacheanus* spider (ca. 6 months old), and one vial with fruit fly food (cornmeal, dextrose and yeast medium). The spiders were left without food for 20 h prior to the trials. We tested male (10 groups) and female (9 groups) fruit flies separately for each experimental and control trial. Each spider was used only once. We did not take into account sex and treatment differences in the body mass of the flies used for the survival study.

### Statistics

We used two-way ANOVAs with treatment (experimental or control) and sex as fixed factors to assess the differences in body mass, lipids, elemental composition, negative geotaxis and survival. We reported only the main effects when no significant interactions between fixed factors were found; otherwise the simple main effects using Tukey HSD were also reported. All statistical tests used in this study were two-tailed. Analyses were performed in Statistica 8.0 for Windows (StatSoft Inc., Tulsa, OK, USA).

## Results

**Figure 1:**
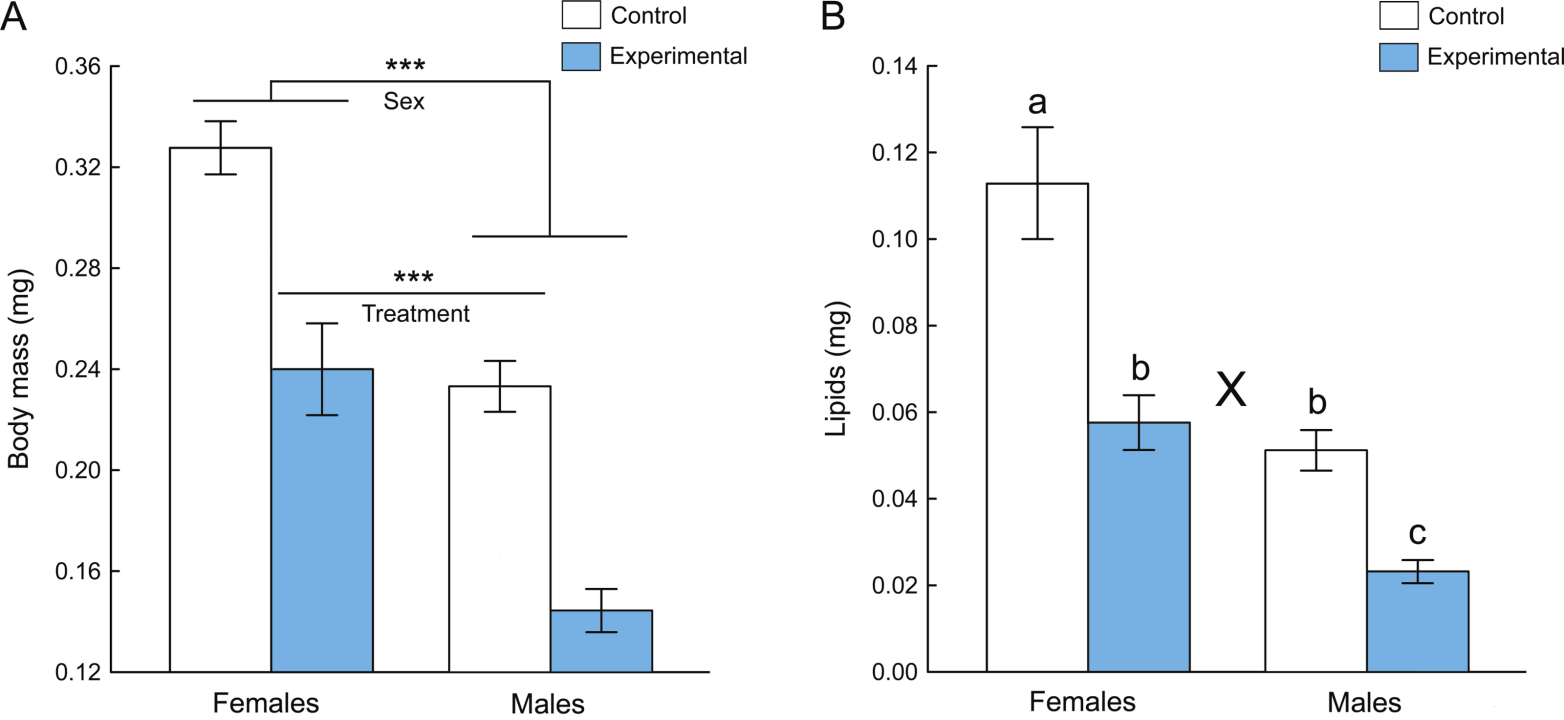
Dry body mass (A) and mass of lipids (B) of *D. melanogaster* flies that were exposed to spider predation in the experimental group and reared without spiders in the control group. Data represent mean ± 95% confidence intervals. *** indicates significant main effects of sex and treatment (two-way ANOVA, *P* < 0.0001). X indicates significant interaction between sex and treatment (two-way ANOVA, *P* < 0.0001); different letters denote significant differences by Tukey’s post hoc tests (*P* < 0.001).

The fruit flies reared with predators (i.e., the experimental group) were lighter than the flies reared in control groups (Fig. 1A), and females were heavier than males (Fig. 1A). Minimum and maximum individual dry body mass was 0.28–0.38 mg (0.32 ± 0.03 mg, mean ± SD) for control females, 0.16–0.31 mg (0.24 ± 0.04 mg, mean ± SD) for females reared with spiders, 0.20–0.31 mg (0.23 ± 0.03 mg, mean ± SD) for control males and 0.11–0.18 mg (0.14 ± 0.02 mg, mean ± SD) for males reared with spiders. However, eclosion time (from egg to adult) did not differ between the control group (9.57 ± 0.79 days, mean ± SD) and experimental group (9.30 ± 0.84 days, mean ± SD; one-way ANOVA: *P* > 0.05). The main effects of treatment (experimental or control) and sex to body size were both highly significant (two-way ANOVA: *P* < 0.0001, Table 1). There was no significant interaction between sex and treatment to body mass (*P* = 0.92, Table 1).

**Table 1:** ANOVA results showing main effects of experimental treatment and sex to body size, energy storage, elemental composition, negative geotaxis and survival of *D. melanogaster* fruit flies. Numbers in bold indicate significant effects (*P* < 0.05), and * indicates significant effects for *F*_(1,76)_.

	Treatment		Sex		Interaction	
Response variable	*F*_(1,96)_	*P*	*F*_(1,96)_	*P*	*F*_(1,96)_	*P*
Body size	220.7	<**0.0001**	256.0	<**0.0001**	0.01	0.920
Lipids	195.6	<**0.0001**	256.4	<**0.0001**	20.6	<**0.0001**
C	0.00	0.97	4.9	**0.029**	3.4	0.070
N	120.8	<**0.0001**	39.3	<**0.0001**	13.2	<**0.001**
C/N	76.9	<**0.0001**	30.1	<**0.0001**	2.2	0.143
Negative geotaxis	787.4	<**0.0001**	59.3	<**0.0001**	0.24	0.625
Survival*	66.6	<**0.0001**	0.01	0.94	0.01	0.94

There was a significant interaction between sex and treatment groups to body fat (two-way ANOVA: *P* < 0.0001, Table 1). Females had more body fat than males in both experimental and control groups (Tukey HSDs: *P* < 0.001). Females in the control group (0.113 ± 0.026 mg, mean ± SD) had more fat than females in the experimental group (0.058 ± 0.011 mg, mean ± SD) (Tukey HSD: *P* < 0.001), while males in the control group (0.051 ± 0.008 mg, mean ± SD) had more body fat than males in the experimental group (0.023 ± 0.005 mg, mean ± SD) (Tukey HSD: *P* < 0.001). Females in the experimental group and males in the control group had similar fat reserves (Tukey HSD: *P* = 0.44) (Fig. 1B).

**Figure 2:**
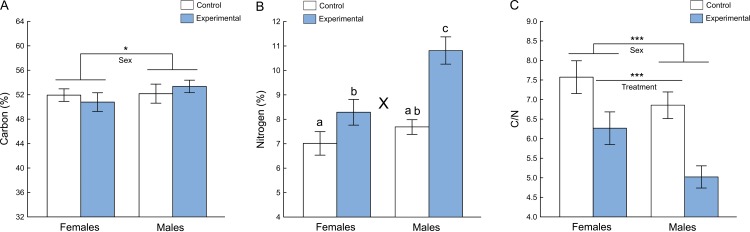
Elemental composition of adult *D. melanogaster* flies reared in different conditions. Average carbon percentage (A), nitrogen percentage (B), and carbon and nitrogen ratio (C) of *D. melanogaster* flies reared with spiders in the experimental group and without spiders in the control group. Error bars represent ±95% confidence intervals. * indicates main effects of sex (two-way ANOVA, *P* < 0.05), *** indicates significant main effects of sex and treatment (two-way ANOVA, *P* < 0.0001). X indicates significant interaction between sex and treatment (two-way ANOVA, *P* < 0.0001); different letters denote significant differences by Tukey’s post hoc tests (*P* < 0.01).

The main effect of sex to body carbon was significant (two-way ANOVA: *P* = 0.029, Table 1). Thus, females had significantly less body carbon than males (Fig. 2A). There was no effect of experimental treatment to the body carbon of fruit flies (*P* = 0.97, Table 1), and there was no significant interaction between sex and treatment to body carbon (*P* = 0.07, Table 1).

There was a significant interaction between sex and treatment to body nitrogen (two-way ANOVA: *P* < 0.001, Table 1). Experimental males had significantly higher concentrations of body N (10.81 ± 1.49%, mean ± SD) than males in the control (7.69 ± 0.85%, mean ± SD) and females in the control (7.01 ± 1.28%, mean ± SD) and experimental groups (8.29 ± 1.29%, mean ± SD) (Tukey HSDs: *P* < 0.001) (Fig. 2B). Sexes did not differ in their body N in the control group (Tukey HSD: *P* = 0.25), and there was no difference in the body N between males of the control group and females of the experimental group (Tukey HSD: *P* = 0.35), while females in the control and the experimental groups differed in their body N (Tukey HSD: *P* < 0.01) (Fig. 2B).

The flies reared without predators (control group) had a significantly higher carbon-to-nitrogen ratio than the flies reared with predators (experimental group) (Fig. 2C). The females had a significantly higher carbon-to-nitrogen ratio than males. The main effects of treatment (experimental or control) and sex to C/N were both highly significant (two-way ANOVA: treatment: *P* < 0.0001, sex: *P* < 0.0001, Table 1). There was no significant interaction between sex and treatment to C/N (*P* = 0.143, Table 1).

**Figure 3:**
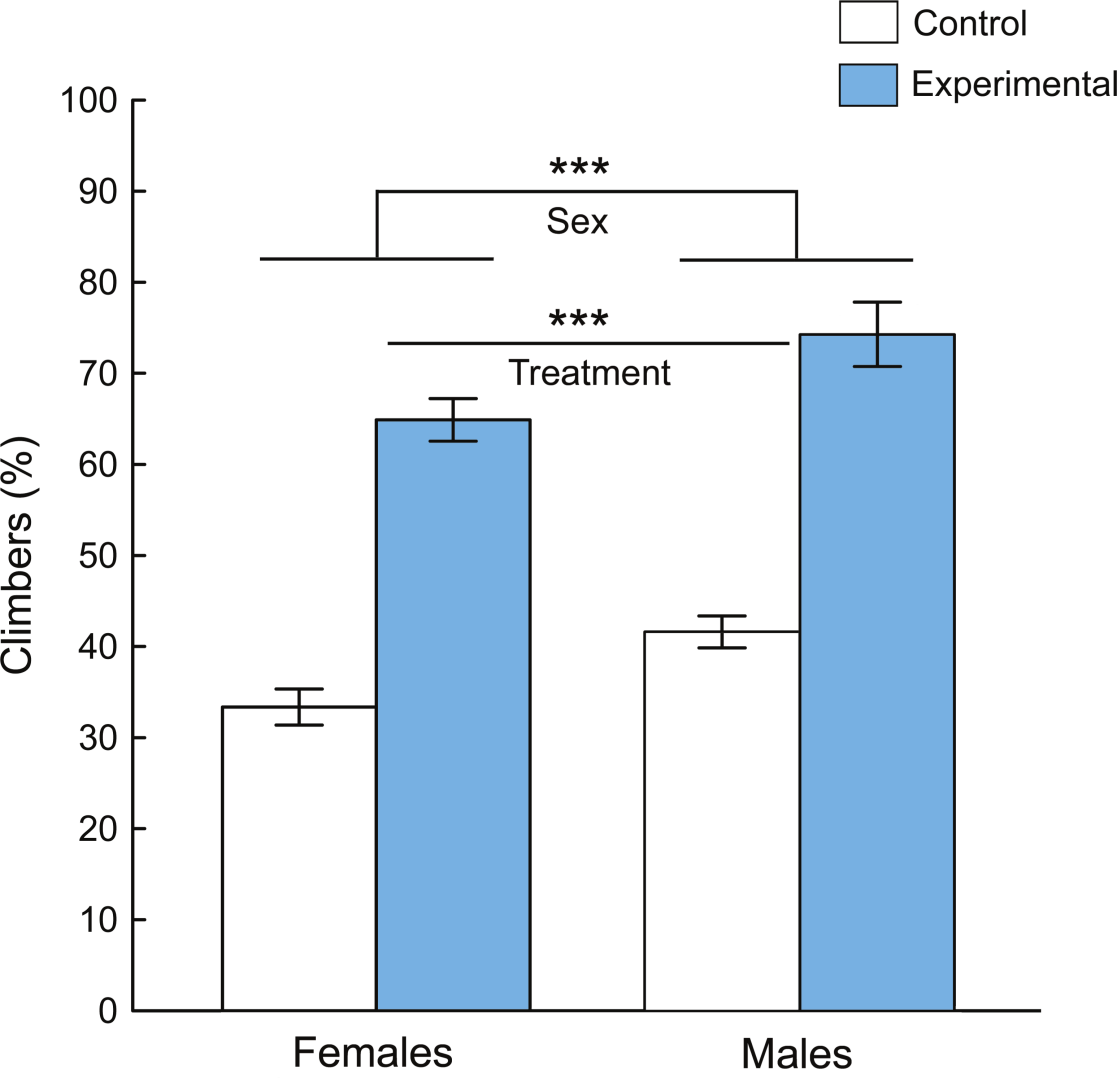
Geotaxis responses (mean ± 95% confidence intervals) of *D. melanogaster* flies reared with spiders in the experimental group and without spiders in the control group. The *y*-axis represents percentage of flies that have reached the 7-cm mark in 10 s. *** indicates significant main effects of sex and treatment (two-way ANOVA, *P* < 0.0001).

The flies reared with predators were significantly faster in reaching the 7-cm mark in 10 s than flies of the control group; and males were significantly faster than females (Fig. 3). The main effects of treatment (experimental or control) and sex to negative geotaxis response were both highly significant (two-way ANOVA: treatment: *P* < 0.0001, sex: *P* < 0.0001, Table 1). There was no significant interaction between sex and treatment to negative geotaxis (*P* = 0.625, Table 1).

**Figure 4:**
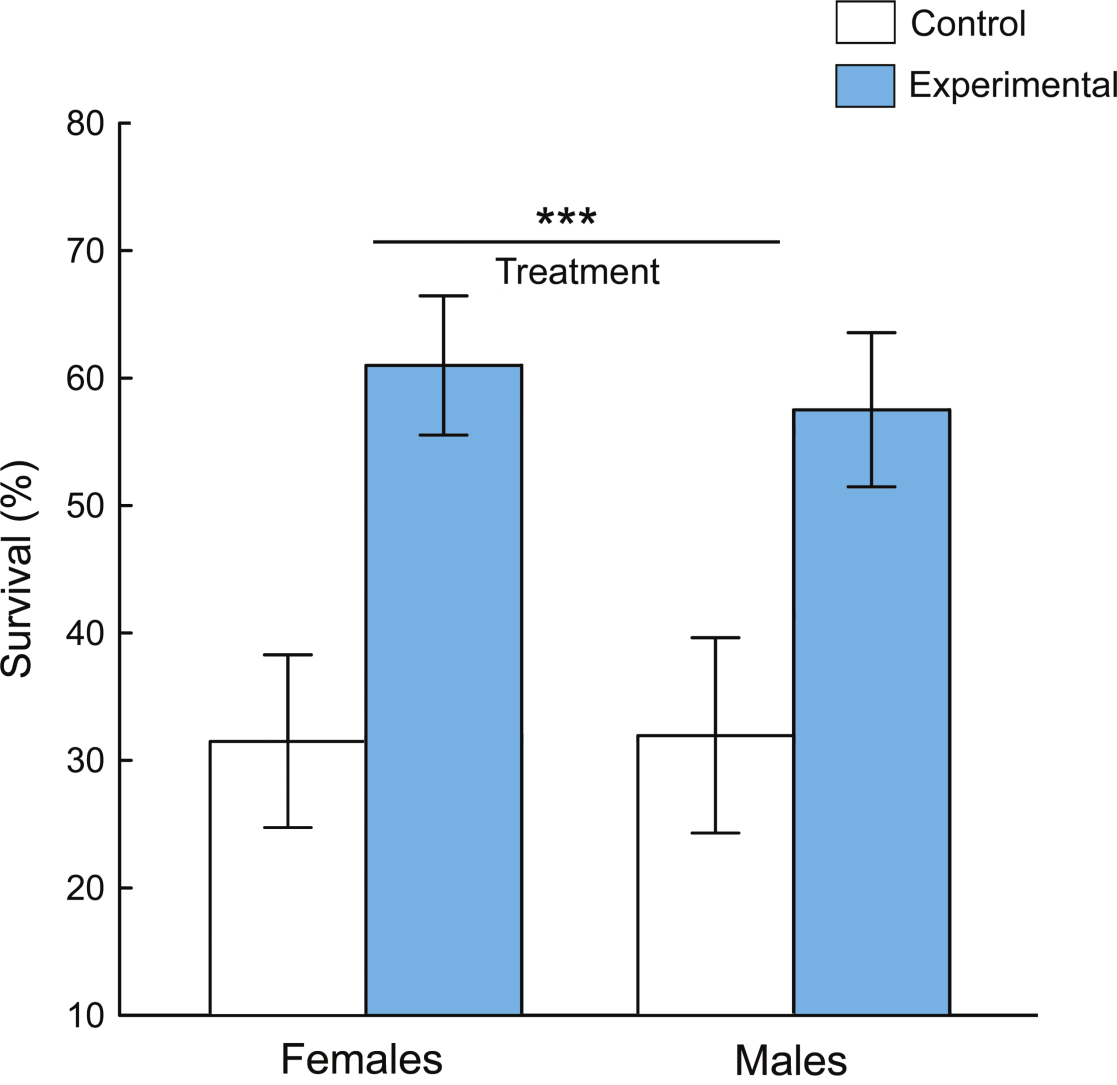
Survival percentage (mean ± 95% confidence intervals) of *D. melanogaster* adult individuals during 12-h exposure to predation by jumping spider. The flies of the experimental group were previously exposed to predation during the larval stage, while in the control group the flies were raised without spiders. *** indicates significant main effect of treatment (two-way ANOVA, *P* < 0.0001).

The flies reared with predators survived significantly better than the control group (reared without predators) (Fig. 4). The main effect of the experimental treatment to the survival of the fruit flies was highly significant (two-way ANOVA: *P* < 0.0001, Table 1). There was no effect of sex to the survival of the flies (*P* = 0.94, Table 1); and there was no significant interaction between sex and treatment to survival (*P* = 0.94, Table 1).

## Discussion

Phenotypic development is the result of a complex interplay involving the organism’s own genetic constitution and the environment it experiences during development, which may contain multiple predators and other stressors (Monaghan, 2008). The estimated mean life span of *D. melanogaster* in field conditions is short, ranging from 7 to 9 days in temperate conditions (Kinross & Robertson, 1970; Corradi & Mourao, 1980), which suggests an important role of predation. The theory of stress predicts that prey exposed to increased predation risk prioritise survivorship over development and reproduction (Adamo, Kovalko & Mosher, 2013). Stress increases mass-specific metabolism (Beckerman, Wieski & Baird, 2007; Slos & Stoks, 2008; Hawlena & Schmitz, 2010a; Hawlena & Schmitz, 2010b), while rising energetic demands increase the overall demand for carbohydrate fuel and lower the need for N-rich proteins necessary for growth (Sterner, 1997). Moreover, stress responses include the breakdown of body proteins to produce glucose (Hawlena & Schmitz, 2010a; Hawlena & Schmitz, 2010b; Christianson & Creel, 2010). Although all spiders in this study were observed preying on *D. melanogaster* larvae, our results do not support the general predictions of ‘fear’ ecology. We did, however, find a significant increase in the body N of *D. melanogaster*, while their body C did not change in response to predation. The finding that males had significantly more body carbon than females after being exposed to predation suggests sex-related differences in response to the risk of predation. Overall, our results indicate that the observed changes in body elemental composition reflect an alternative strategy as to how animals cope with stressful environments during ontogeny. This shows that the strategy discussed earlier, i.e., adaptive decreases in body nitrogen and increases in body carbon in order to mount more efficient anti-predator responses (Hawlena & Schmitz, 2010a; Hawlena & Schmitz, 2010b), is not the only possible ontogenetic response to prey-induced stress, although it utilises the same mechanism of adaptive body composition changes. Evidence shows that exposure to predation risk triggers sustained psychological stress (Clinchy, Sheriff & Zanette, 2013). However, effects other than depressive ones have also been predicted and observed (Peacor, 2002; Boonstra, 2013). We found that *Drosophila* fruit flies reared under conditions of predation risk attained adulthood with lower body mass than fruit flies raised without predators (Elliott et al., 2016). This suggests a trade-off between developmental speed and somatic growth (Stearns, 1992). Growth and development comprise multiple semiautonomous units that may compete for resources, which is why the growth of some traits constrains the growth rates of other traits. It is important to note that large body size is usually preferable due to several complementary reasons (Nakazawa, Ohba & Ushio, 2013; Blanckenhorn, 2000), such as an advantage in intra-specific competition (Griffiths, 1992), increased fecundity (Gilbert, 1984; Leather, 1988) and extended survival (Gilbert, 1984; Griffiths, 1992). However, we found that the flies reared with spiders not only had smaller body mass, but they also climbed faster in negative geotaxis tests and survived better under the actual predation by spiders. Higher concentration of body N may be linked to investment in protein production, which results in greater muscle mass and higher climbing speed or better manoeuvrability while escaping predators. Lower body reserves or body lipid content may also contribute to survivorship by significantly increasing body mass-dependent escape ability in insects (Almbro & Kullberg, 2012). The flies under predation risk may adopt a fast life history strategy (which accounts for their small size), and so future studies should investigate whether flies under predation risk utilise resources more adaptively in reproductive effort rather than somatic growth.

Differences in body N, lower lipid reserves, higher climbing speed and better survival of the fruit flies reared under conditions of increased predation risk indicate that developmental strategies change early in life. This can, furthermore, be attributed to developmental plasticity in order to improve survival after the predators were introduced into the environment. Thus, environment not only constrains but also guides, or even induces development in *Drosophila* fruit flies (Gilbert, 2001; Gilbert, 2005). However, it is not clear why grasshoppers (Hawlena & Schmitz, 2010a; Hawlena & Schmitz, 2010b) and fruit flies responded to predator presence in such a different way during ontogeny. Perhaps this may be explained by differences in the duration of their development: while *D. melanogaster* needs around 10 days from egg to adult at 25°C, grasshopper development often lasts for months. Stunted behavioural responses induced by stress may improve the chances of reaching adulthood in grasshoppers because shy/stressed individuals have a greater probability to survive than their bold counterparts under conditions of elevated risk (Krams et al., 2013a; Krams et al., 2013b). In fruit flies, development might be fast enough to avoid chronic stress caused by predation, while acute stress may improve an animal’s survival in life-threatening situations (Dhabhar & MeEwen, 1997), suggesting that the changes in body elemental composition observed in this study can be evolved and adaptive (Boonstra, 2013).

Another explanation for the observed differences between grasshoppers and fruit flies might be linked with developmental differences. *D. melanogaster* is a holometabolous insect, meaning that it has a larval and a pupal stage prior to the adult stage. Grasshoppers are hemimetabolous insects with several nymph stages preceding adulthood (Gullan & Cranston, 2005). In most holometabolous insects, life cycle prevents larvae from occupying the same ecological niche and lessens the potential competition between adults and larvae (Speight, Hunter & Watt, 2008). In contrast, intraspecific competition among different age classes and developmental stages of hemimetabolous species such as grasshoppers can be high, which brings excessive stress especially to subordinate individuals such as grasshopper nymphs. If environmentally induced stress causes differences in body elemental composition and other responses between hemimetabolous and homometabolous insects, further studies should find lower body N and higher body C in hemimetabolous insects not only in individuals exposed to a high predation risk. This would also apply to other stressful conditions such as development under high conspecific densities and severe competition for food with other nymphs and adults.

The basic idea of ‘ecology of fear’ is that predation risk induces stress which increases metabolic rate and shifts nutrient demand from proteins (which are crucial for organismal growth) towards carbohydrates that fuel the heightened respiratory demands of anti-predator responses (Stoks et al., 2016; Trussell, Ewanchuk & Matassa, 2006). In their experiments with grasshopper development under the risk of spider predation, Hawlena & Schmitz (2010a) gave the grasshoppers a choice between two diets differing in protein and carbohydrate content. They found that grasshoppers preferred carbohydrates when predation risk increased. In the present study, fruit flies underwent body composition changes *without* being given a choice between diets, which shows that organismal body composition can change independent of diet composition. To explore this phenomenon in more detail, a possible avenue for further research would be to analyse the differential flexibility of predator-free and predator-exposed fruit flies to switch between diets rich in proteins and carbohydrates, and how this affects their body elemental composition. The importance of this type of research is heightened by recent findings that revealed the existence of dynamic compensatory mechanisms that allow larvae of the tobacco hornworm caterpillar (*Manduca sexta*) to increase assimilation efficiency and extraction of N from their food when vulnerable to predation (Thaler, McArt & Kaplan, 2012).

In conclusion, we showed that predation has an effect on the phenotypic development of *D. melanogaster*. Importantly, the larvae of *D. melanogaster* responded to predation risk in a way that was not predicted by ‘ecology of fear.’ Developmental speed and/or species-specific features of metamorphosis are the most likely possibilities for explaining the observed differences between *D. melanogaster* and grasshoppers (Hawlena & Schmitz, 2010a; Hawlena & Schmitz, 2010b). A number of previous studies found that insect life histories are extensively affected by habitat type, temperature, food availability and its quality (Speight, Hunter & Watt, 2008; Sinclair et al., 2003; Hawlena & Schmitz, 2010a; Hawlena & Schmitz, 2010b; Krams et al., 2015). If we wish to fully understand the life histories of insects and the limits of their developmental plasticity, developmental strategies need to be studied as the interplay of ambient temperature, nutritional and energetic value of food, and competition within and between different developmental stages of the same species in the presence of predators. Predators affect the development of their prey; they can be found virtually in every community and ecosystem, and their influence appears to be more significant than previously thought (Stoks et al., 2016). The present results highlight the importance of predation pressure on *D. melanogaster* ontogeny.

##  Supplemental Information

10.7717/peerj.2314/supp-1 Supplemental Information 1 Drosophila body characteristics and negative geotaxis under spider predation Data on dry body mass, lipid amount, nitrogen & carbon concentrations and climbing speed during negative geotaxis trials in Drosophila fruit flies (males, females) reared with spiders (predator identity) and in the control group (reared without predators).

## Additional Information and Declarations

### Data Availability

The following information was supplied regarding data availability: Open Science Framework: https://osf.io/jxpwy/.
